# Supplementary material for: Towards unified AI-driven fracture mechanics: the extended deep energy method (XDEM)
Source: Nat Commun. 2026 Aug 15;17:8492. doi: 10.1038/s41467-026-76748-1 (PMC13484471; doi:10.1038/s41467-026-76748-1)
Supplement: Supplementary file 3 [file 41467_2026_76748_MOESM3_ESM.zip › pic/Results/Crack_propagation/Straight/3D_dis_force/Tension_3D/Loss_convergence_6.pdf]

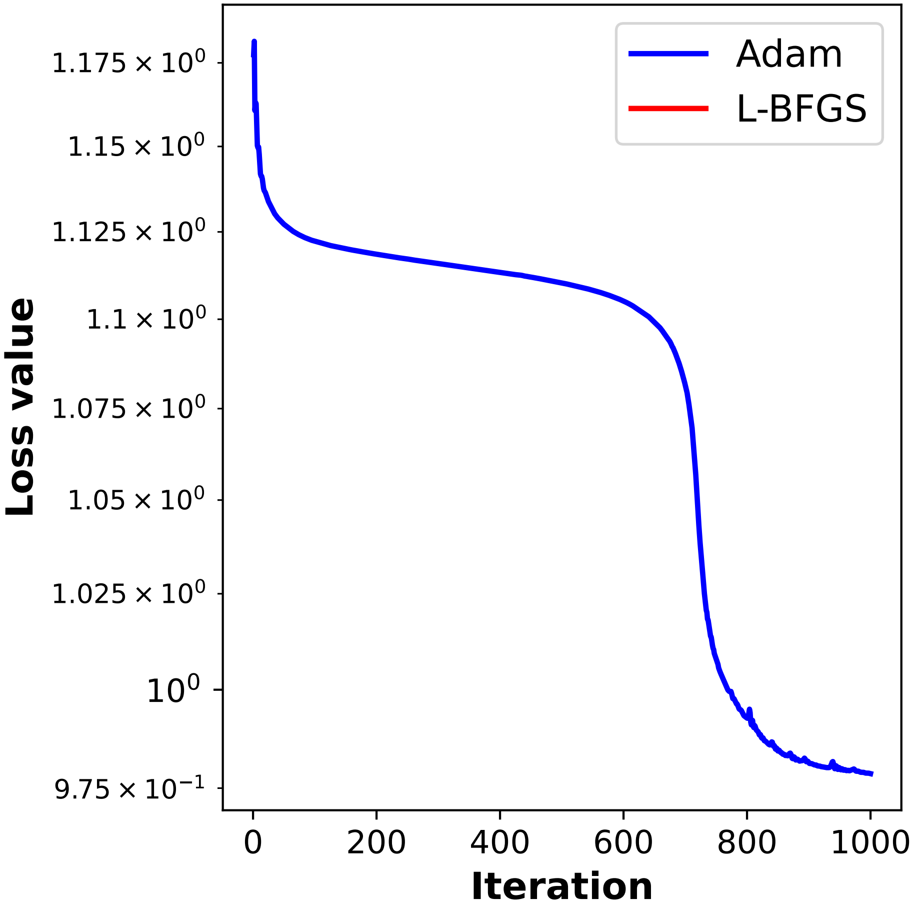

Adam
L-BFGS
Loss value
Iteration
1.175 × 10⁰
1.15 × 10⁰
1.125 × 10⁰
1.1 × 10⁰
1.075 × 10⁰
1.05 × 10⁰
1.025 × 10⁰
10⁰
9.75 × 10⁻¹
0
200
400
600
800
1000
